# Spatial video geonarratives and health: case studies in post-disaster recovery, crime, mosquito control and tuberculosis in the homeless

**DOI:** 10.1186/s12942-015-0014-8

**Published:** 2015-08-08

**Authors:** Andrew Curtis, Jacqueline W Curtis, Eric Shook, Steve Smith, Eric Jefferis, Lauren Porter, Laura Schuch, Chaz Felix, Peter R Kerndt

**Affiliations:** Department of Geography, GIS Health and Hazards Lab, Kent State University, #413 McGilvrey Hall, Kent, OH 44242 USA; Department of Geography, High-Performance Computing and GIS Lab, Kent State University, #407a McGilvrey Hall, Kent, OH 44242 USA; Geography, Department of Social Sciences, Missouri Southern State University, 3950 E. Newman Road, Joplin, MO 64801 USA; Department of Social and Behavioral Science, College of Public Health, Kent State University, Kent, OH USA; Department of Criminology and Criminal Justice, University of Maryland, College Park, MD USA; Gould School of Law, USC, Los Angeles, CA USA; Tuberculosis Control Program, County of Los Angeles Department of Public Health, Los Angeles, CA USA

**Keywords:** Spatial video geonarrative (SVG), Geographic information system (GIS), Global positioning system (GPS), Narrative, Context, Post-disaster recovery, Crime, Mosquito control, Tuberculosis

## Abstract

**Background:**

A call has recently been made by the public health and medical communities to understand the neighborhood context of a patient’s life in order to improve education and treatment. To do this, methods are required that can collect “contextual” characteristics while complementing the spatial analysis of more traditional data. This also needs to happen within a standardized, transferable, easy-to-implement framework.

**Methods:**

The Spatial Video Geonarrative (SVG) is an environmentally-cued narrative where place is used to stimulate discussion about fine-scale geographic characteristics of an area and the context of their occurrence. It is a simple yet powerful approach to enable collection and spatial analysis of expert and resident health-related perceptions and experiences of places. Participants comment about where they live or work while guiding a driver through the area. Four GPS-enabled cameras are attached to the vehicle to capture the places that are observed and discussed by the participant. Audio recording of this narrative is linked to the video via time stamp. A program (G-Code) is then used to geotag each word as a point in a geographic information system (GIS). Querying and density analysis can then be performed on the narrative text to identify spatial patterns within one narrative or across multiple narratives. This approach is illustrated using case studies on post-disaster psychopathology, crime, mosquito control, and TB in homeless populations.

**Results:**

SVG can be used to map individual, group, or contested group context for an environment. The method can also gather data for cohorts where traditional spatial data are absent. In addition, SVG provides a means to spatially capture, map and archive institutional knowledge.

**Conclusions:**

SVG GIS output can be used to advance theory by being used as input into qualitative and/or spatial analyses. SVG can also be used to gain near-real time insight therefore supporting applied interventions. Advances over existing geonarrative approaches include the simultaneous collection of video data to visually support any commentary, and the ease-of-application making it a transferable method across different environments and skillsets.

## Background

This methods paper advances a spatial approach to capture and analyze “lived experiences”, which previously and primarily had been an undertaking of qualitative research. This paper adds to a confluence of epistemologies and geospatial methodologies exploring the potential of qualitative approaches such as the narrative or interview in place, with geospatial technologies, geographic information systems (GIS) and global positioning systems (GPS) to enhance our understanding of how people, places, and the relationships between them shape a range of outcomes from environmental conditions to health [1–6].

In the public health and medical communities, need for personal context is compelling; to understand the day-to-day activity, challenges and emotions of a “patient” in order to improve education and treatment [7, 8]. Connected to this is a growing body of research suggesting local expert and community member knowledge sources are required to understand the context of health [9–13].

From a spatial scientist’s perspective, the challenge is how to collect these data in a robust and transferable way to allow for data collection, analysis, and theory development [14] while at the same time being easy to operationalize. Although awareness of the complexity inherent in capturing geographic context is important, and should be reflected in methodology that systematically collects and analyze these data [12, 15, 16] it is also important to be inclusive and not alienate groups due to overly complex or technical techniques. After all, if we want to know *why does this happen here and not there*, or, *who or what are we missing here*? we need a method that can be employed across multiple environments with only moderate technical ability. This paper presents such a method, the spatial video geonarrative or SVG. SVG is an environment-inspired dialogue or commentary; it is a simple yet powerful concept—to have those most impacted describe what it is like to live in the area under investigation. Others have used qualitative GIS [17], mixed methods [18] and even narratives [14, 19, 20] to enrich more traditional spatial analytical approaches. SVG as proposed here is an advance over similar approaches for two reasons, video and ease of use:

1. SVG is captured using spatially encoded video which allows for post-collection comparison of the narrative with a visual of what was being discussed. In addition, video for the same space from previous time periods can be used to complement reflective comments about the past. Finally, the video can be a data source in its own regard, with visual objects being mapped out to support the narrative.
2. The data collection, manipulation, mapping and analysis are straightforward requiring only basic GIS skills. This ease of use makes SVG a possibility for different locations, different topical areas, and for different levels of expertise. The SVG output can still be analyzed using more sophisticated qualitative or spatial analytical approaches, but it is not a necessity. For the method described here, all equipment is off-the-shelf, and the vast majority of all data manipulation requires only basic spreadsheet, GIS and Google Earth skills. A short piece of computer code with one upload button, and one time stamp input window is the only additional technology required.

The utility and flexibility of the SVG method will be illustrated through four different case studies. These case studies are not designed to be mini-research papers with a typical research question, methods, analysis and results framework, but rather each will show the potential of what might be achieved. It is left to the reader to extend the research thought process within each example, and at the same time drawing parallels to other topical areas and environments. The paper will conclude with sections describing the next steps in the SVG evolution; spatially analyzing words as a point data source, the logistic and psychological challenges of SVG, and issues of ethics and spatial confidentiality associated with the method.

## Methods

SVG is an advance on other geonarrative approaches as it adds a visual component, the spatial video, which can be used as an additional contextual resource when investigating (and mapping) the commentary. Video are collected, usually using an automobile, with a latitude and longitude coordinate stream being simultaneously attached to the video stream. The post-processed spatial video can be thought of as similar to Google Street View, a spatio-visual reference which is gaining in popularity as a source for digitizing information into a GIS [21–23]. Unlike Google Street View, SVG data collection is in the control of the researcher. The spatial video “kit” utilizes an off-the-shelf extreme sports camera (Contour +2) with an inbuilt GPS. The camera has associated free software (Storyteller) for displaying the video and video path. Having access to this free software is important as the GSV method should be a collaborative tool not only allowing for local expertise to be mined, but also giving researchers, activists, or concerned individuals a “voice” in how their local health situation is monitored and analyzed. Indeed, of the four case studies presented here, three involve local collaborator collected data. Past spatial video projects have been well documented with topics ranging from post-disaster damage and recovery [24, 25], mapping the built environment in association with the crime-health nexus [26], and most recently, to identify street-scale health risks in Haiti [27]. In addition, the team has utilized this technology in multiple other countries, including Bangladesh, Belize, Cambodia, China, Kenya, Liberia, Malawi, Tanzania and Zambia. For this paper we will focus SVG examples based in the United States. Although spatial video has been collected using a variety of modes (car, motorbike, bicycle, boat and by foot), the most frequent approach is mounting four cameras to the windows of a vehicle. The following seven steps provide the basic procedure for most SVG.

### Spatial video

Two to Four Contour +2 cameras are positioned around a vehicle using suction window clamps.^a^ For maximum clarity these cameras point out above a rolled-down window. In environments perceived to be more sensitive or dangerous, the cameras can be mounted on the inside of the window for unobtrusive data collection. High definition video means that the car can drive at road speed as a later paused image is clear enough to provide a suitable digitizing source. Multiple cameras mean that there is compensation in case one video recording on either side fails, and if three of the four GPS fails. Digitizing information from the video into Google Earth is appealing as both Contour Storyteller (the software available with the camera) and Google Earth share the same imagery [27]. The camera needs no outside GPS antennae, and is turned on by sliding the rocker bar forward. A series of lights flash to give a status update on the battery, micro SD card, and GPS fix. The battery typically lasts for 2 h, but can be extended to six if connected to a car charger. On full HD mode the 32 GB card can record for approximately 4 h. Some pre-GPS work is required to make the camera fix onto satellites quickly. For example, making a pre-fieldwork connection to Contour Storyteller allows for the software to be updated with satellite positions, and turning the camera on some time before the ride helps with locking in.

### Geonarrative

Commentary is recorded directly by the cameras, or (preferably) onto a digital recorder with an output from that device being split and fed into each camera. The camera contains an internal and external microphone. These can suffice if the cameras are close to the subject, and if video is being recorded through the window. If the vehicle is large, loud or has a separation between the front and back seats (such as a police car) then an external microphone is used. The Contour +2 does not provide power through its microphone input connection, so a powered external microphone is required. The best setup is having the subject hold a microphone which has an output cord going through a splitter and then input into each camera. It is vital that a sound can be matched on the external microphone and the video in order to interpolate words over the GPS path.

### Data collection

The typical ride lasts between 45 min and 3 h. The “team” includes a driver, a camera operator (usually in the back seat), and the subject sitting as a front passenger.^b^ During the ride, the driver and the camera operator help facilitate the interview by asking prompting and clarifying questions. The ride starts with the reading of an IRB-approved permission sheet. Depending on the project, there is a collection of basic socioeconomic and subject histories (especially how long he/she has lived in the area being collected), and a perceptual map exercise. This is useful to start each ride in the same way, to get each subject in the same mindset, and as a future research project of comparing perceptual maps to SVG content.

### Data download

On the culmination of the ride, data are downloaded from each camera either by plugging the device into a computer or extracting the micro SD card. Once downloaded, two post-processing stages occur: renaming all video files and the creation of a metadata sheet. The size of video output files from the Contour +2, especially in Full HD 1040p mode, is approximately 1 GB per 9 min. The camera software breaks the video stream into approximately 36 min segments. After the ride, each of these segments is renamed according to the date, the side of the camera, and the sequence of the video file within the total number of segments.^c^ The GPS from each video segment is extracted using Contour Storyteller (as GPX) and each file is imported into Google Earth. By saving all video sections for one camera as a “save place as”, a KMZ is generated of the entire route. This can be imported into ArcGIS 10.2 so that each path (the number of paths equals the number of cameras) can be compared for performance.^d^ A metadata sheet is created in word processing software and contains an image of the route map exported from the GIS, a brief summary of the ride (notes on the subject, about the commentary, and any technical problems), the name of each camera and its position on the car, and an assessment of the video, audio and GPS quality for each camera. These sheets are an imperative companion to the archived video, allowing for easy reference in future projects. Metadata sheets are also important for SVG analysis as they facilitate combining the best audio and GPS source.

### Transcription

The SVG audio is transcribed using any software of choice. Although this can be done in Microsoft Word, for example, specialist software allow for easy time stamp insertion and easy control over moving the audio back and forth. The final output must include six numbers (hours, minutes, seconds) separated by colons. Time stamps precede any substantive comment, usually every sentence, and may even be inserted before key spatial locations, as long as each starts a new text row. As the specially designed software interpolates words between time stamps, having a stamp close to an important statement, and followed by a second stamp will help with mapping words to the precise location. Time stamps must be sequential, and duplicates are not allowed^e^.

### Spatial word interpolation

The time stamp of a key sound on the audio recording is matched to the audio on the video source allowing for the “real” time stamp of the audio to be established. Again using the export GPS function in Contour Storyteller, but this time using a CSV format, coordinates and their associated Greenwich Mean Time (GMT) can be displayed in Excel. This also gives the first time where a GPS fix occurred, which may be different from the beginning of the video media time. It is important to match sounds on the recorder and the video after this fix has occurred. The High-Performance Computing and GIS Lab (HPCGIS) at Kent State University has developed computer code (from this point G-Code) that interpolates the transcription across the route and calculates the “real” time stamp of each spoken word. G-Code requires the transcribed commentary to be uploaded in .txt, and the associated GMT time input for the beginning of the file. All words occurring between time stamps are interpolated across the number of seconds between time stamps. If there is a problem in the transcription, for example two identical time stamps, the code stops processing and identifies the time where the problem occurs. After the .txt file has been edited to remove all errors, and G-Code has successfully run, the output of three columns (ID, time, word) is copy and pasted back into Excel. Excel manipulation is required of both the CSV and G-Code output to ensure that the GMT time stamp column in both is ready to be joined together in a GIS. Manipulation includes saving both files as .prn files, then reimporting them so that the columns of text are appropriately separated.^f^

### Mapping words

Both the spatial video file (CSV manipulated into an Excel format) and the G-Code output (saved as an Excel file) are added to the GIS. The spatial video file is added next, with latitude and longitude plotted to produce the data collection path. The G-Code excel file is added and then the new route shapefile is merged with this using a table join (order is important as each word needs a coordinate). This merged file is plotted using the latitude and longitude columns, and the word column is displayed as a label for each point. At this point the SVG can be mapped, manipulated, queried, and analyzed like any other GIS point file.

There now follows four case studies showing the utility of this approach with minor method manipulations detailed depending on the subtleties of the project.

### 1. Mapping perishable data: the recovery and psychopathology nexus after the 2011 Tornado in Joplin, Missouri

*“I didn’t go there very often, I’ve been there since they’ve rebuilt but I’m always gonna remember there because that unfortunately is where I saw the first people* – *the first time I saw people were killed in the tornado in a car up there.”* – *SVG Participant*

Researchers have previously considered the geographic perspective of the health-hazard nexus [28], though the role of micro spaces is still largely undeveloped. Spatial video has previously been used to match mortalities to damage patterns, and then to monitor spatial patterns in recovery linking these to potential social outcome data including stress and crime [26, 29]. If we focus primarily on the recovery phase of the disaster cycle, then manifestations of poor health in the literature largely concern psychopathology [30]. Of these, what few spatial studies exist tend to miss patterns at the micro spaces scale primarily due to data availability [31]. SVG is a method that can mine the connection between how events during and after the disaster affect and continue to affect day-to-day activities, *in a spatially specific manner*. To illustrate the potential of this new research direction, a series of SVG were conducted in Joplin, Missouri primarily during 2014 using an unstructured interview format where the subject described any tornado related event with only minimal questioning and clarification. The resulting narratives were transcribed, and imported into G-Code before mapping in ArcGIS 10.2. Just as with the opening quote to this case study, many of the comments are directly related to the night of the tornado. However, the conversation often hinted at the psychological burden still being carried. Although it is possible to overlay multiple SVG to create a new data layer of the common experience, for an extreme event such as this, there is also value in the single experience as an indicator of the types of challenges and stresses felt while moving through recovery. In this regard, although specific locations can be mentioned (the corner where the bodies were seen) it is more useful to think of the insights as spatially “fuzzy”.

Comments can be used to gauge the spatial heterogeneity of recovery, or as in the following example, what it was and is like to live in the aftermath of a disaster.

*“Well, for a while it was just deserted. And our home was* – *we have a new home there. Within a little over a year after the tornado. But it was one of the only structures around. It was dark and it was deserted. I’d say within another year there was a bunch of more substantial rebuilding, and still going on. But I’d say a disproportionately high number of homes there are Habitat for Humanity homes inhabited by people who did not live in this area before the tornado.”*

If we reconsider the previous literature on psychopathology, the SVG not only supports existing theory (evidence of triggers, negative or positive coping mechanisms) but also illustrates how the situation of the individual, both during the disaster and recovery phase, is related to health outcomes and the need for spatially targeted intervention. It would be interesting to see how disaster psychologists could further mine these data, or this data collection approach, in their research.

*“In the course of a normal day, I’ll always find something that reminds me of it.”*

#### Method variant

**Fig. 1:**
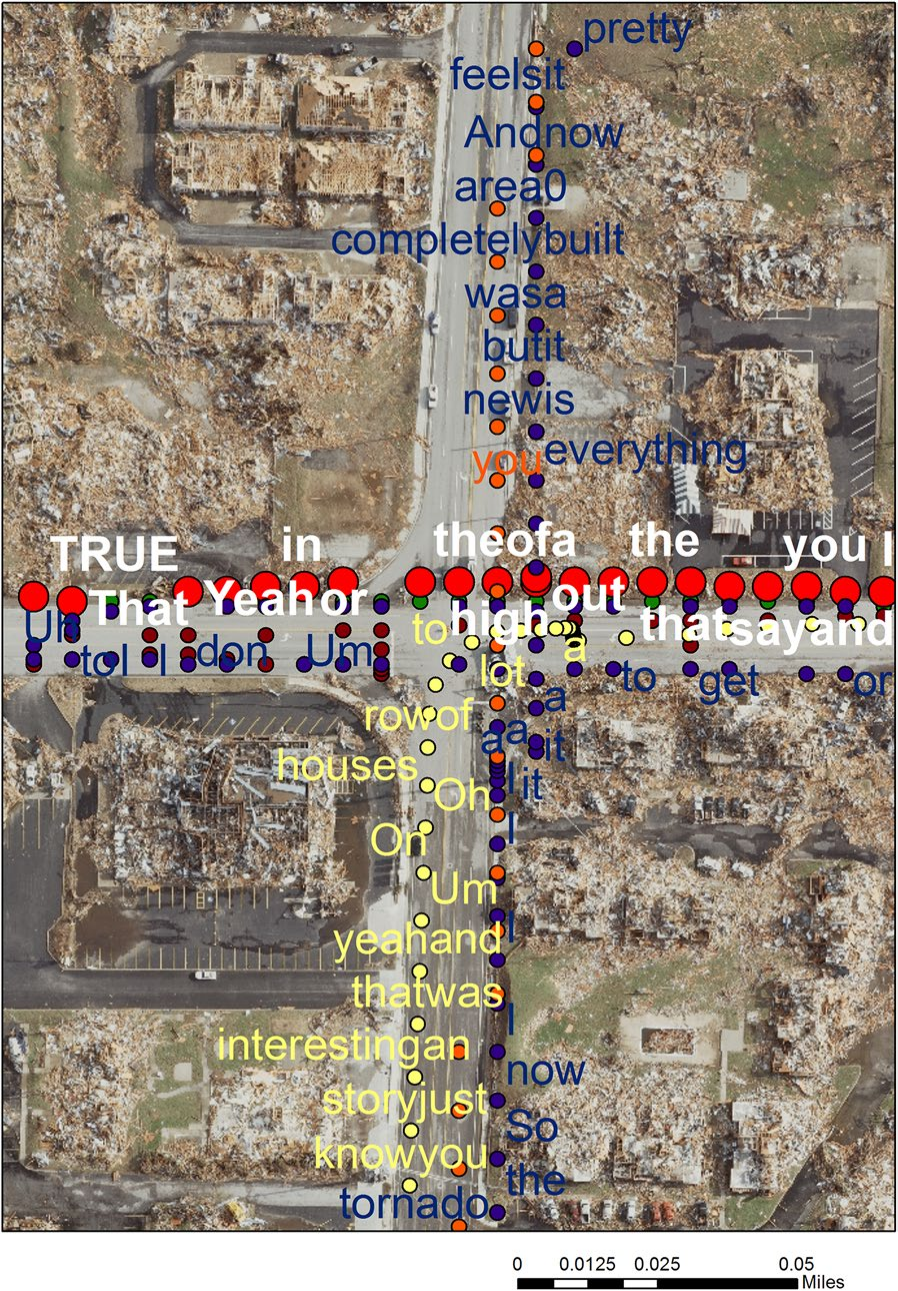
Six different SVG intersect (each a different *colored* point) at a key location on the tornado path where multiple mortalities occurred.

Six different SVGs were uploaded into the GIS. One manipulation included using a key word query to identify the locations of important phrases, such as “recovery”. By using the time stamp associated with each word coordinate, it is possible to return to the original transcription to gain further context of what was being described. Another easy task is to go to an area of importance, such as a particular street segment, to see what was described at that location. In Fig. 1 six different SVG intersect (each a different colored point) at a key location on the tornado path where multiple mortalities occurred. Although it is hard to read this graphic as whole, it should be thought of as a SVG index. By zooming into a location such as this, each GPS point, or displayed word, can be selected and matched back to the original transcription. Here the full commentary can be read and questions asked such as how do different people comment on this location, how many refer back to the events of the day, and how many comments focus more on recovery or the current setting. For any of these queries or manipulations, the associated video for that ride can be accessed to see exactly what was being described.^g^

### 2. Mapping contested spaces: multiple perspectives for the same geographic area

*“It’s reduced crime, but it’s because it’s reduced the life, it’s thinned it out. And urban renewal is urban removal.”*- *SVG Participant*

Another compelling reason for conducting multiple SVGs is to assess how perspectives on a neighborhood may vary. This has implications with regards to understanding a health problem, and developing subsequent interventions. For example, it is widely acknowledged that there remains a disparity in birth outcomes for African Americans especially in large urban areas—a situation which has not really changed in the last two decades. Mapping these disparities often only verifies what any local health worker already knows [32]. The challenge is to “contextualize” data, since a spatial hotspot analysis only tells a partial story—it identifies a (statistically significant) concentration of events without providing explanation or causation [33]. Research might validate some of the patterns identified, but limitations still exist with the type of data available (for example linked infant birth and death certificate data). Many practitioners would argue that there is invaluable insight to be gained from those who live and serve the hotspot areas, if only it can be accessed and analyzed.

Although a limited number of SVGs, as in the Joplin example, can be illuminating, this constrains the analytical possibilities. Having multiple perspectives provides a more holistic impression, and through triangulation [34], offers validation with regards to the places, spaces or ideas that are identified. The opening quote to this case study displays one perspective on the dichotomous responses regarding a classic “contested space” in Akron, Ohio. A city initiative in the mid-2000s removed a large number of blighted homes from the neighborhood. From a police perspective the move was a success. For some residents, the neighborhood itself has been winnowed, both physically and socially,^h^ with overall crime being reduced as a result of the associated reduction in population, though more specific and localized hotspots remain. This changing urban setting generates several other problems for local area health professionals, such as the high rate of domestic disturbance call-outs, many of which occur with children being present (this involvement of children can be extracted from Akron Police Department data). As such, this neighborhood is an example of the health-crime nexus found in many inner city areas; high levels of violence (and child exposure to violence), high injury rates, high chronic disease rates, substance abuse and as suggested by the domestic call outs, a high level of family stress [35]. Although intervention is needed, what form should it take? Indeed, although we have outcome data, what are the actual perceived problems or pathways? SVG can capture professional insight (such as police officers), local experiences or emotions (residents and clergy), and by overlaying and comparing these perspectives, help formulate a geographic and socially relevant intervention.

The approaches as presented in the first case study can also occur with the SVGs collected here. For example, one particular Akron Metropolitan Housing Authority (AMHA) complex in this neighborhood displays as a hotspot for crime, family disturbance, and different health problems. Although the spatial analysis of call-outs or health outcomes would identify this as a geographic area of concern, the narrative itself sheds light on the complexity involved:

*“They just have drama here…Some folks will be here for a while and then get kicked out, so there’s a zero drug policy and zero visitor policy. So like there will be a single mother but the baby daddy stays and they’ll find out the baby daddy stays and they’ll kick him out.”*

#### Method variant

**Fig. 2:**
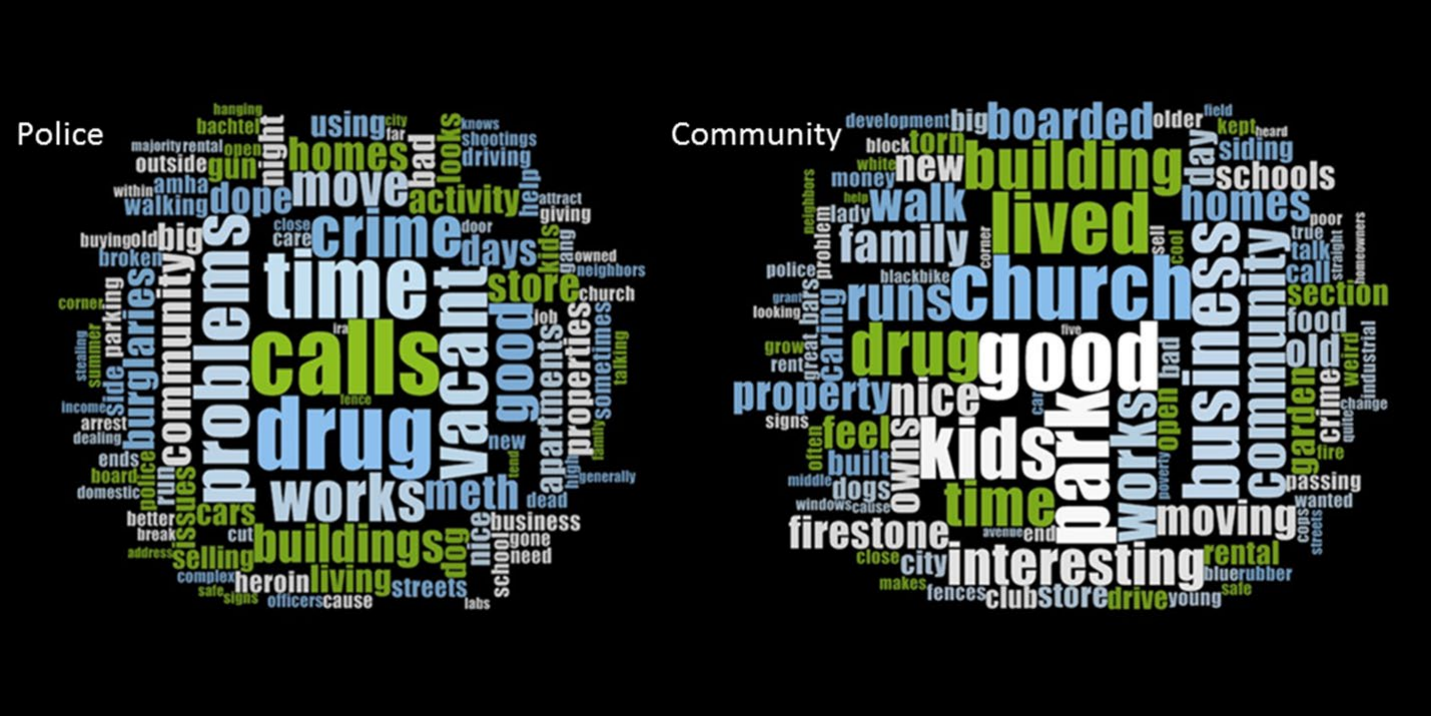
Word clouds constructed from the SVG for police and community members.

Over 30 SVGs have been collected for the subject neighborhood, with the G-Code mapped paths being overlaid to form a composite impression of the neighborhood. With a larger number of SVG, different approaches are required to search through text for key themes or “nodes” in addition to commonly accepted terms such as “drugs”. This can be done using more sophisticated software, such as NVivo, or even a simple word cloud.^i^ For example Fig. 2 displays word clouds constructed from the SVG for two main groups: residents and police. What is immediately obvious is the different way the area is described, which is understandable given the overseer role of the police while residents (generally) focus on the positive aspects of their home space. Just as with the Joplin example, any word from these clouds can be queried and then mapped, either for a single SVG, or in total for all subjects. The combined SVG maps can be spatially analyzed using a variety of different point pattern techniques (the key word is the numerator, all words are the denominator) to find statistically significant concentrations of a phrase.

**Fig. 3:**
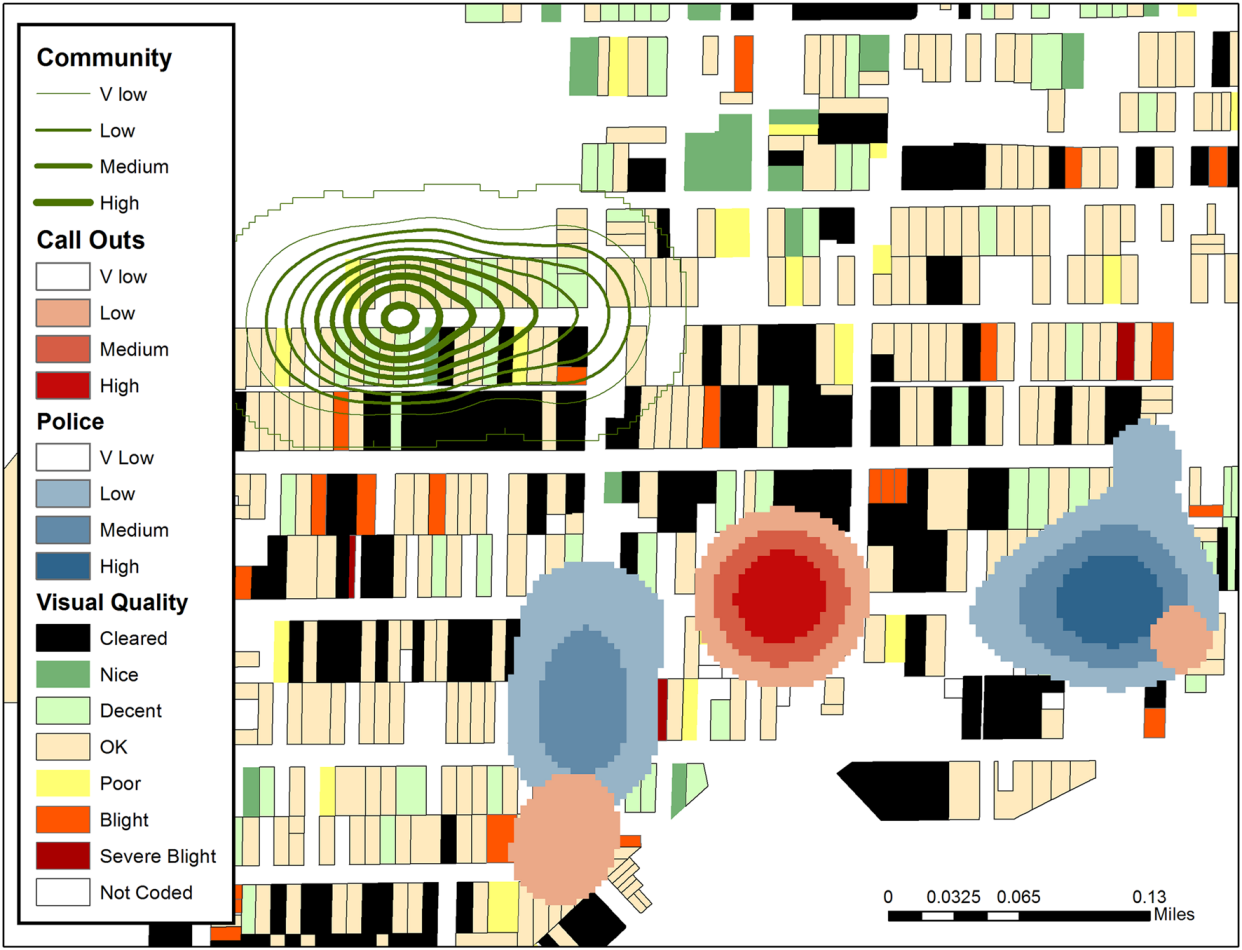
Kernel density estimations (KDE) of all mentions of SVG “meth” by community members (*green*) and by the police (*blue*). As a part of the overlay, police calls for service are shown in* red* and output from a spatial video built environment survey are displayed for each parcel.

Consider Fig. 3 which uses kernel density estimation (KDE) to display all mentions of SVG “meth” by the police, and then by community members. All drug “call outs” by the police are also displayed as a comparison with an official incident data layer.^j^ The example KDE intensities are overlaid on properties which have been visually assessed and mapped using the spatial video as a source.^k^ Even from these three comparative layers we can see that community knowledge differs from both the police and from actual response calls, especially for one street segment which resonates across the community SVG. The visual quality of buildings can be used to help explain why these patterns exist. For example, on the street identified as problematic by community members, little building clearing has occurred. However, there are also no examples of blight with only two residences being classed as “poor”. Imagine the additional insight gained by adding more traditional health data, such infant health outcomes, to these maps.

### 3. Mapping “institutional knowledge”

(00:14:00) Prior to closing this up, I could stick my dipper in there, you know a foot to a foot and a half.

(00:14:10) I used to set my traps up here but I haven’t caught anything when I set it up here.

(00:14:17) But if you go over here.

(00:14:33) And if I set it up over here, it’s pretty moist and there’s probably water inside.

(00:14:41) And in this basement area right here I put a trap in here and I catch Aedes and Culex in this area right here.

The focus of the SVG can be to capture the knowledge of health professionals who “work” an area by spatially capturing their experiences. The danger of not recording this knowledge is that when a key employee leaves then most of his/her knowledge disappears. On a recent SVG with an environmental health specialist focused on lead exposure in Akron, Ohio, this point was raised. Although the initial response was that the next specialist would soon achieve the required certification, during the ride it became evident to the interviewee *as she listened to her own comments* that she had amassed a vast knowledge of local area risks, solutions, and especially atypical problem solving. One example being the ability to visually assess a property to gauge the likely lead threat posed. At the end of her SVG the first temporal layer of an institutional knowledge map had been established. Now a new worker could re-watch the video and listen to her narrative.

This approach is also beneficial if more than one professional covers the same area as each learns the same on-the-ground information separately often with little ability to share knowledge. Capturing health-related institutional knowledge is not limited to social health situations. As an example, consider the following SVG for mosquito control for a mid-sized urban area.^l^ The vector control team had been diligently controlling mosquitos for many years. Different environments pose different mosquito “problems”, and for this settlement, the biggest challenge was seasonal rains and the type of drainage system in situ. Other important considerations were the efficacy of different mosquito control strategies, the proximity to vulnerable populations (especially daycare centers), and where positive disease cases (of West Nile virus) had been found. One of the reasons for capturing the SVG, beyond establishing a record of control approaches, was an impending concern of Dengue and Chikungunya, both diseases having been found within 100 miles.

#### Method variant

Just as with the first two case studies, G-Code was used to map out every word from a day-long drive around the study area that covered all past and present mosquito trap locations. Different key words important to mosquito control such as a specific mosquito species were mapped. However, as the purpose here was to create a map of institutional knowledge, the narrative was re-read for all commentaries that had significance to mosquito control, and could be directly tied to a location, such as a building or field. These segments of text were then rejoined back to the mapped text in the GIS (again using the GMT stamp) so that the coordinate of the first word became the anchor for that comment. Further spatial manipulation occurred if the point was in reference to a specific location such as a building by editing the SVG coordinate onto the shape as identified by comparing the spatial video with high resolution aerial photography in the GIS.

**Fig. 4:**
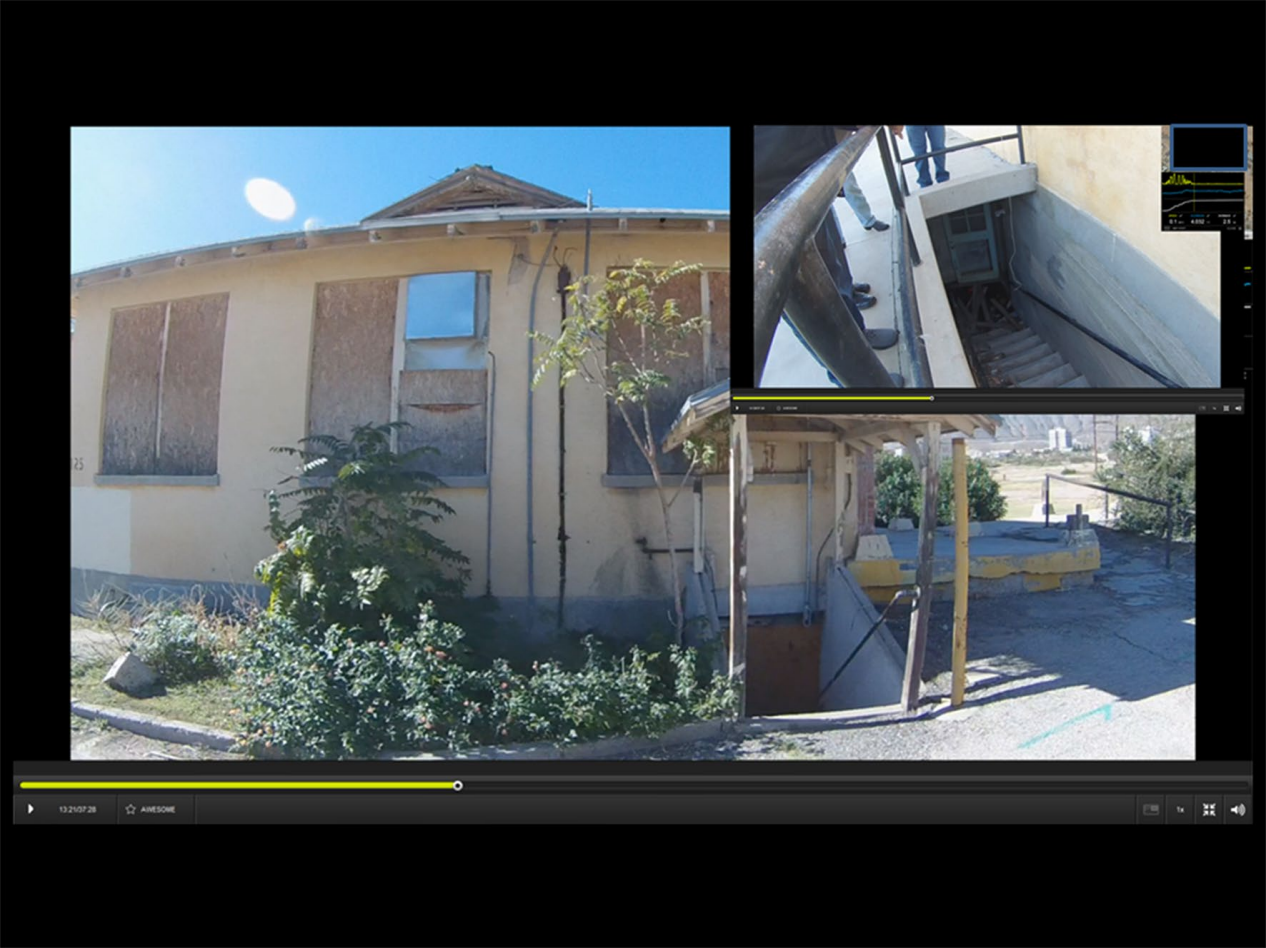
Snapshot images from both a vehicle and then hand held SVG to capture institutional knowledge.

Figure 4 displays the abandoned building references in the opening quote of this case study. The figure also displays the software used to view the video, which normally shows the associated map in the inset box, though it is covered here with the second still to preserve locational anonymity. Two examples are chosen; the main video still is from the vehicle as the team stops at this building known for mosquito problems. The inset still is when one camera has been removed and the team walks the premises to talk about the exact location of the problem areas and traps, in this case the flooded section of the basement is visible. Unlike with the first two case studies the Contour +2 camera was removed from the window mount and hand carried along with the microphone. As long as neither the camera nor audio recorder is turned off, the same SVG method applies.

**Fig. 5:**
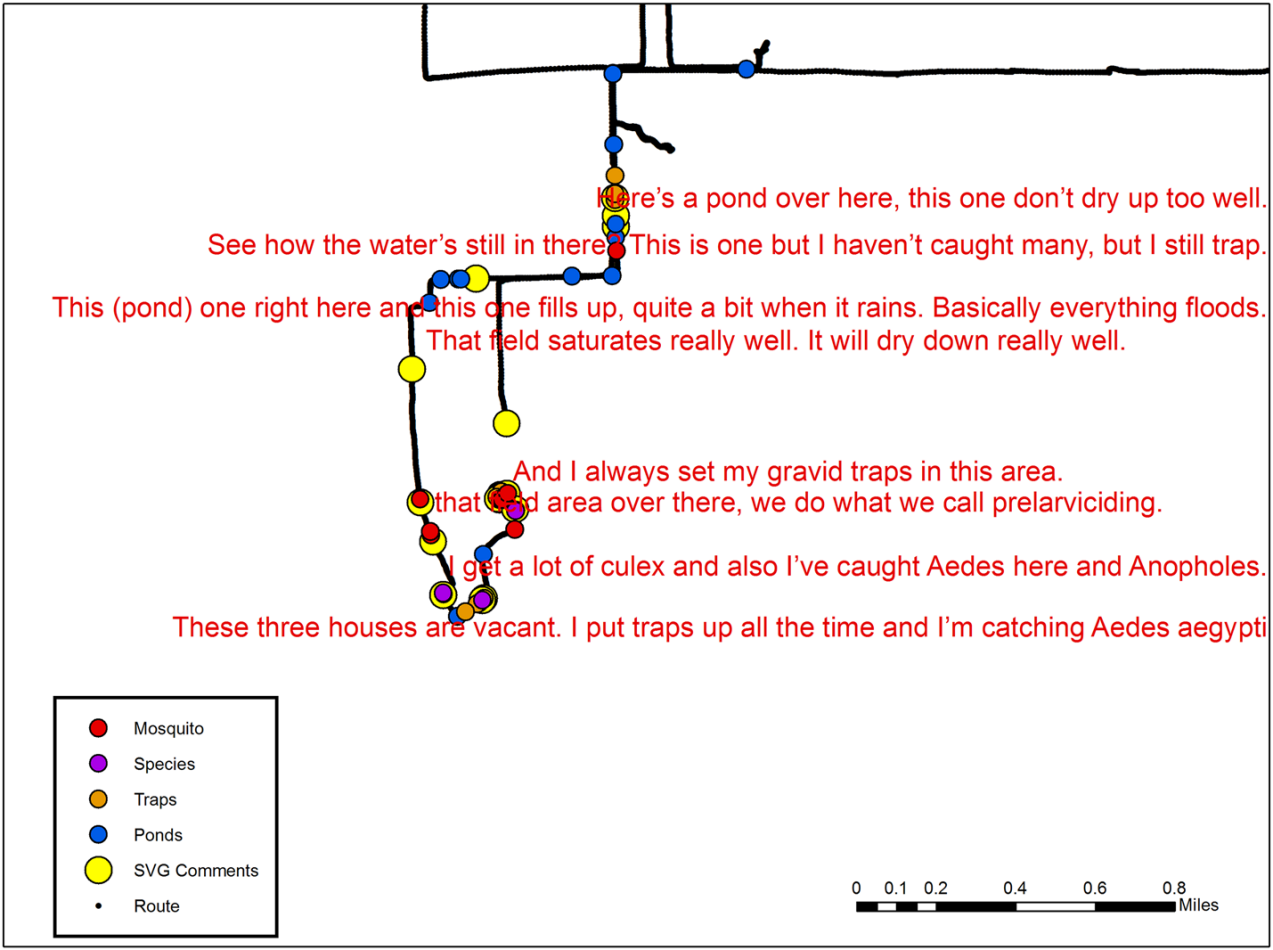
A section of the SVG institutional knowledge reference map before translation into Google Earth.

As the main purpose of collecting an institutional knowledge SVG is as a resource or archive for the professional organization, it is important to have a ubiquitous means of dissemination. To do this, point commentaries were exported to Google Earth. This resulted in a series of pins that would reveal the relevant commentary for that location in a format that could easily be emailed to any associated worker. A section of the reference map before Google Earth translation is seen in Fig. 5. All spatial information has been removed at the wishes of the mosquito control board. Apart from the example statements that are tied to a specific location, key words have been extracted from the G-Code, allowing for the easy mapping of any trap location, or for a specific mosquito species. Just as with Case Study 1, these data are more limited for analysis. However from a practical perspective, reviewing these Google Earth maps at the beginning of each season could help with strategic planning. Repeating the exercise at each season’s end would also grow the institutional archive. The archive is further enriched by the spatial video, with each comment or insight being accompanied by the actual image, as seen in Fig. 4.

### 4. Mapping the homeless in Los Angeles

(00:07:34) So, you’ve got different types of homeless, which I know it sounds really crazy, but the guys that are on this side of the freeway,

(00:07:40) are definitely a little bit, um, rougher around the edges, than the people,

(00:07:47) on the other side of the 101. And the reason why is because you’ve got MacArthur Park,

(00:07:51) you’ve got like, more of the suburbs, you’ve got more areas where, um the hills.

(00:07:57) Um here, it’s concrete jungle, and they’re completely trapped.

(00:08:03) So you’ll see them, their like, their sixth senses grow a little bit harder. Their vibe is a little bit more intimidating.

(00:08:09) It’s just because they’re in the middle of the jungle. That’s my impression.

(00:08:17) When I first started coming down here, you would never see families with kids.

(00:08:22) When you go to the jewelry mart, you’d never see families with kids. You would see crack deals.

**Fig. 6:**
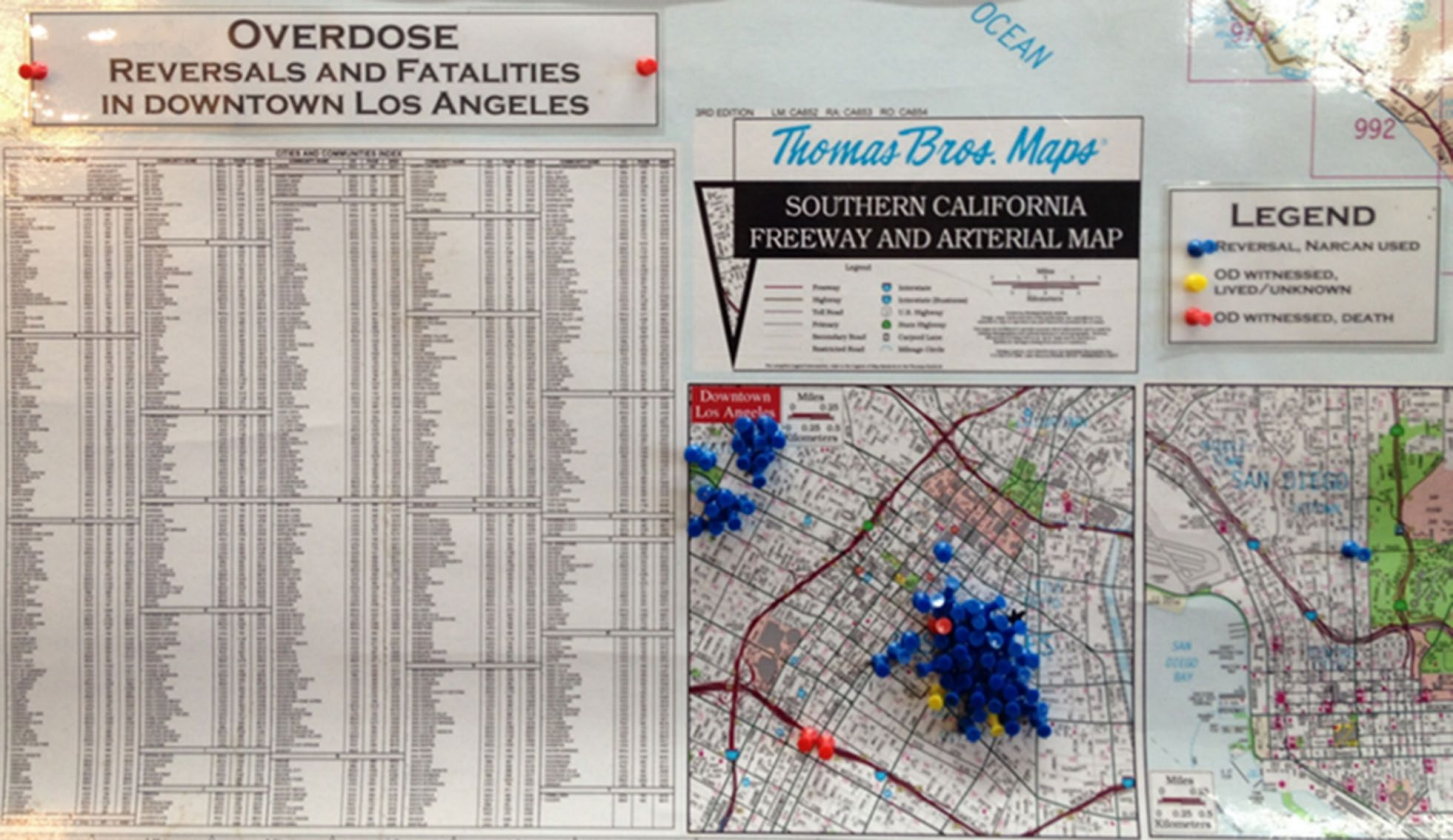
A literal pin* map* showing the application of Narcan medication located in a non-profit medical facility in skid* row*. The *map* which was photographed on the wall of the facility shows the scale of the problem, but none of the other attribute information about each victim is recorded.

One of the biggest geospatial health challenges in the United States is how to capture health compromised cohorts with no typical address [36, 37]. Two examples are the “homeless”^m^ and sex workers. The former by definition have no address, while for many sex workers there is no constant abode, often moving between temporary locations. Both groups have high health risk factors and we would expect them to show as hotspots if these cohorts are captured in normal surveillance data. As reliable spatial data are not available, we are left with three challenges: it is hard to know where spatially high concentrations of a disease occur, and who are at risk in primary proximate locations. Secondly, we have no idea as to the activity patterns and daily mobility paths that might help explain or predict disease spread. Linked to this issue, we have only a limited understanding of how and where best to intervene. Tuberculosis and sexually transmitted diseases are perpetual health concerns associated with the homeless and sex workers in Los Angeles, and yet there is a dearth of understanding about these cohorts, especially from a spatial perspective (Fig. 6).

Mapping proxies for the homeless have been tried, such as discarded drug needles [38]. Just as with the three previous case studies, SVG provides a novel alternative to help explain current homeless spatial patterns, and as an insight into what it is like to be homeless. Spatial video can be used to help map visible physical locations, while SVG can capture individual experience, contested spaces, and institutional knowledge from those who serve these areas. Two ongoing spatial video and SVG projects in Los Angeles have focused on knowledge of health outreach professionals south of downtown regarding “spatially challenged cohorts”: the homeless in skid row and sex workers. Consider the following SVG conducted in Skid Row. A longer section of narrative is provided to help illustrate the spatial richness of many SVGs.

(00:20:16)[DR] How come there is no one on the other side?

(00:20:18)[HW] Sun

(00:20:19)[DR] Oh the sun

(00:20:21)[HW] So they basically fort, I mean it’s a lot of work to be homeless. You have to find the right spot,

(00:20:26)[HW] you have to make sure there’s no sun, you have to make sure that people aren’t going to kick you out,

(00:20:30)[HW] you have to make sure you’re not coming on someone else’s block. You have to make sure like.

(00:20:36)[HW] It’s a lot of work.

(00:20:39)[DR] And are they, are, you said people are loners, are some people actually group, gregarious and connected to each other or do they look out for each other?

(00:20:50)[HW] yea there’s a sense of community.

(00:20:53)[HW] There’s a sense of community for sure. But you also have to keep in mind, what drugs are they using?

(00:21:00)[HW] because and what time of the month, what time of the month is it. Are they going to be sharing their drugs in order to have friends,

(00:21:09)[HW] Are they going to be just doing all the dope themselves, …. the time of the month, matters a lot.

(00:21:17)[HW] See there are too many gates here

(00:21:20)[DR] What do you mean too many gates?

(00:21:21)[HW] Too many gates, it’s not a safe place to hang out.

(00:21:26)[DR] Because too much traffic?

(00:21:29)[HW] Too much traffic, too much activity.

(00:21:33)[HW] She’s probably looking for a rock.

(00:21:45)[HW] So I want to go, so you see how that’s safe there?

(00:21:52)[HW] That’s a safe spot.

(00:21:53)[HW] uh huh, that’s a safe spot.

(00:21:57)[HW] So as I said, when we first started, we were on Fifth and Main and we came here in two thousand five.

(00:22:41)[HW] And this is kind of where it starts.

(00:22:46)[DR] Do all those people camp together? Do they know each other?

(00:22:49)[HW] Mhmm

(00:22:58)[HW] See they respect Skid Row Housing Trust, they’re not on that corner.

(00:23:04)[HW] But now they’re over here.

(00:23:33)[HW] I mean that alleyway there’s a lot of stuff that goes on. A lot of stuff that goes on because you’ve got these guys on the corner, they’re slinging, they’re hustling.

(00:23:46)[HW] They know what’s up. You’ve got the watchdog, you’ve got the old man, you’ve got the one who’s carrying it and you can tell by their shoes.

(00:23:54)[DR] Who the dealers are, who’s dealing?

(00:23:55)[HW] Yea. You can tell by how clean their shoes are.

(00:24:03)[HW] How white their shirts are

(00:24:08)[HW] Do you see what I mean, do you get the vibe?

(00:24:13)[DR] So is this where the alleys are to the left?

(00:24:17)[HW] The alley is right here to the left.

(00:24:19)[HW] And this is always where they comingle.

SVG captures location specific and more general information with regards the homeless. Insight is gained into the micro spaces of where the homeless set up camps, as well as specific places of risk and safety. There is also more general temporal information with regards to the social cohesion—drug nexus. Personal experience also reveals the micro space indicators of drug activity.

#### Method variant

**Fig. 7:**
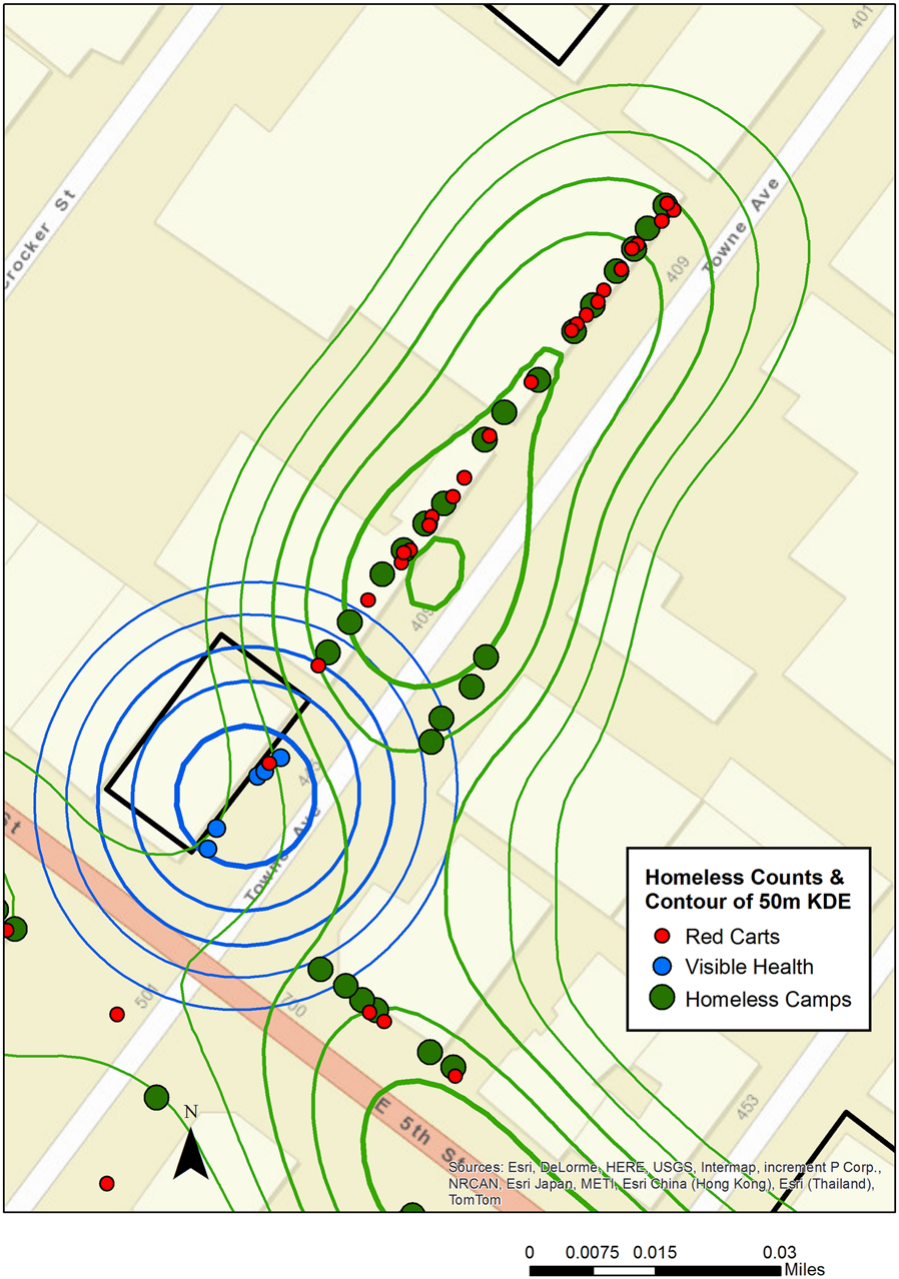
Locations of homeless camps, *red* shopping carts, and people with visible health problems such as a wheel chair.

The spatial video is used as the primary data source with visual aspects for Skid Row being digitized either into Google Earth and then imported into the GIS, or digitized directly into the GIS. Consider Fig. 7 which displays the locations of homeless camps, red shopping carts which had been bought for the homeless,^n^ and people with visible health problems such as a wheel chair. Each location represents an object digitized from the spatial video and then visualized using contours from a 50 m KDE. Questions that arise from these maps, such as why is there spatial variation in the location of camps, can be investigated using the approaches described in the first case study. Insight into the homeless lifestyle can be gained either by using the map and selecting the word path on the map for the area of interest, or by going to the SVG and finding relevant sections of text, in this case that reveal the camp location decision making process.

It is easy to imagine how these types of insights can help inform intervention strategies. By repeating spatial video runs over multiple time periods, homeless camp stability can be assessed and even proxy addresses given to the residents. On top of these layers, SVG provides invaluable insight into the daily activity patterns of the homeless. At the time of writing, another TB outbreak has occurred in this general vicinity. Imagine if SVG is used as part of a surveillance strategy whereby interviewing those infected, becomes part of a standard approach to identifying activity spaces, mobility patterns and camp locations.

## Discussion

Spatial video geonarratives provide an excellent tool to collect/create data where none exist, or to add context to the spatial analysis of more traditional data. The SVG case studies presented here are “typical”, with a subject accompanying the researcher in a vehicle while narrating the passing landscape. Variants can include walking tours or other modes of transport such as boats.^o^ An alternative approach to field SVG is using a controlled setting, the most simple of which is sitting in front of a computer screen which displays a video of the environment. A narrative can be captured just as in the field, the commentary being recorded along the whole video length, or typed with comments being tagged by the associated video time stamp. There are different reasons why this controlled setting testing is appropriate, most notably being the safety of the subject if he/she prefers not to be seen in the neighborhood.^p^

From a topical perspective there is a host of different health problems that can be investigated with SVG; any situation where a fine scale insight can help provide context or guide intervention, and especially if change occurs over time. Likewise, SVG should be considered for any situation where institutional knowledge is valuable and perishable. However, we fully acknowledge this is a nascent methodology which offers exciting possibilities, but still has multiple aspects needing research attention. For example, how should we analyze these data, what are the best psychological strategies for information extraction, and the issue of ethics and spatial confidentiality.

There are several methodological considerations with a SVG. After processing and mapping a SVG using G-Code, each word receives a coordinate. However, there are several decisions that need to be made during the transcription process that will affect the final mapping. Have any abbreviations been used in the transcription? Have all voices been transcribed? Are there sections not relevant to the SVG, such as at the beginning during the setup? Manual input decisions need to be made with regards to text selection for mapping. For example, when mapping “drugs”, does this include both spatial and non-spatial mentions? Does it also include the driver/interviewer? An easy solution may be to only use text from the subject with a specific spatial mention. As with any spatial analysis, the prudent course is varying the inputs into the analysis to assess the stability of results.

A second spatial question includes “spatial fuzziness”. Even with a specific mention, such as the alleyway in which drugs were being taken, how close is the mapped word to the actual location? Three factors are relevant here: which word is used in the mapping (the first in the statement, the word “alley” or “drug”), the speed of the vehicle, and whether the subject prefers to talk about a location before or after, but not proximate because of a fear of the location.^q^ More research is needed on how frequently specific locations are mentioned regarding the distance *before* and *after* a place once a subject is visually cued.

If we move to the analysis of the text, especially mapping hotspots of context, questions arise with regards to which words to map. A simple solution is to use all variants of a key word, such as “drug*”. However, should we include other words relevant to drugs (crack, meth), even though some (meth) have distinct geographies, and arguably a different meaning (a meth house and drug dealing are a connected part of the same problem but display different social and spatial locational nuances). Unfortunately, many health-related phrases are even more “fuzzy”. For example, rarely will a subject use a term such as “infant mortality” so finding associated phrases is important. Although such libraries and dictionaries of words and phrases are available to connect text to perceptual processes, emotions, and cognitive processes [39], especially connected to mining social media [40], SVG arguably pose different problems and as such require new thinking eventually leading to guidelines.

If SVGs are to be used as a new data source, questions also arise as to how to define an appropriate denominator—is it the total number of words, or a minimum number of rides, or a minimum number of the same spaces (street segments) covered? Should common words such as “the” or “it” be removed?

Another area of future research involves the “art” of interviewing. Ideally, a subject will take instructions at the beginning of a ride and completely let the landscape cue all comments. The reality is that the interviewer will insert comments with regard to clarification (of subject matter and spatial location), to ask follow-up questions on a topic discussed, to direct the conversation towards the goal of the study, and to break pregnant pauses. There is an established literature on interview techniques, including the perils of framing in oral histories^r^—but how translatable are these guidelines to SVG? Researchers discussing the potential of SVG have commented on the need to use a semi-structured approach to ask the same questions of all subjects. However, is this correct for SVG if we hope the landscape will guide the comments? If it is correct, to what degree would the same question, asked at different locations with different visual stimuli result in a different response? And how should such directed questions be controlled for in any spatial analysis of the text?

Finally, as with any geospatial advance, it is imperative researchers consider the confidentiality and ethical consequences. Typical Institutional Review Board concerns for social and spatial scientists involve preserving the confidentiality of the subject, and making sure he/she feels safe during the interview. However, how should we disseminate what is said and then mapped? SVG maps might reveal the identity of the subject through mapping their “story”. These maps may also reveal sensitive information about others in the narrative. There are still relatively few rules governing the spatial display of sensitive data [41] and there are no guidelines for such second-person information mapping. If a house is identified (and mapped) as having had multiple health problems, then ethically we have to consider such information as sensitive and adhere to normal spatial confidentiality conventions, even if IRB does not direct us to do so. Other ethical considerations arise; what if a house, or alley, is identified in multiple SVGs as being problematic. What are the implications to those who live there (or nearby)? What of local business owners? Will these maps negatively influence their trade? Will house prices drop? Should we report that mosquito traps near certain day care centers have previously had West Nile virus positive mosquitoes? It is sometimes difficult to predict these spillover impacts so the suggestion is again to consider all SVG mapping as confidential and use widely accepted cartographic approaches to mask any data that can be reengineered. Even so, spatial confidentiality and SVG is a topic that needs to be considered further, if only to illustrate possible pitfalls and provide informational support to IRB.

A final note regarding ethics is, should we be videoing the streets? This is a commonly voiced concern with regards to Google Street View. The typical solution is to blur faces. Typical researcher-collected SVG will not be displayed publically on websites. As long as care is taken in publication and presentation graphics, are we correct in assuming we have a right to video our neighborhood streets?

All these topics need additional attention; however, these should not halt the possibilities offered by SVG. Especially as spatial video and SVG are excellent participatory tools that can involve people or groups integral to a problem but who normally have no voice. By allowing “collaborators” to collect their own data, and be able to see and hear their own video and through SVG identify important content, hopefully steps are being made to break down the academic-community divide.

## Conclusions

From a topical perspective there is a host of different health problems that can be investigated with SVG; any situation where a fine scale insight can help provide context or guide intervention. Likewise SVG should be considered for any situation where institutional knowledge is valuable and perishable, or simply where data do not exist. In many ways, limits of use are only imposed by the creativity of the researcher. However, we fully acknowledge this is a nascent methodology and although it offers exciting possibilities, enthusiasm has to be tempered as there are multiple aspects of SVG still needing research attention, especially concerning ethical implications.

## Endnotes

^a^The Contour Plus 2 camera has a fixed wide-angle (Aspect ratio 16:9, Field of View 125 degrees, high definition (1920X1040)) lens which is excellent for coding visual attributes into a GIS.

^b^An additional team member can ride in the back at the risk of overwhelming the subject, and getting in the way of the equipment (there are several cords running into the backseat including power and audio splitters, and having too many people on the backseat can cause these to come unplugged).

^c^An example video file name would be: LA031015RA1of4. This video was recorded in Los Angeles, on the 10^th^ of March 2015, on the right side, and there were 4 total segments.

^d^Alternatively the GPX can be brought directly into the GIS and manipulated in that software.

^e^If comments are made by two people in quick succession, a second must be added or subtracted to give separation.

^f^The current version of G-Code is available on request from the corresponding author, or Eric Shook, code developer. A future web based system is planned that will remove the Excel data manipulation stage.

^g^The benefit of having multiple time period spatial video is that the map becomes an archive. It is easy to go back in time to the actual damage that occurred at the location in Fig. 1 to give a visual to the memory described. Of course this requires a spatial video to have been collected soon after the disaster.

^h^In addition, key aspects of the social fabric, such as the neighborhood middle school were also removed.

^i^In their paper Kwan and Ding (2008) incorporate more sophisticated qualitative analysis of narratives in their 3D-VQGIS GIS based qualitative analysis software.

^j^Police call out data includes 911, non-emergency calls, and even police patrol generated call-ins.

^k^The authors have developed different visual coding strategies for the built environment stemming from their early spatial video disaster-recovery work.

^l^Although no sensitive information was revealed in the SVG, at the wish of the mosquito control board all locational identifies removed.

^m^Here we generalize the term to mean those who literally live on the street.

^n^See http://articles.latimes.com/2011/aug/08/opinion/la-oe-dietrich-justice-20110808 for the challenges faced by the homeless with regards safeguarding their possessions.

^o^Walking narrations have been used in the United States in association with “walk to school” initiatives, and in several international settings especially for slum settings in Bangladesh and Kenya.

^p^There is also no reason why narration couldn’t be recorded as part of a virtual classroom experience, or as a means to crowd source map making, and as a spatio-visual focal point for multiple expert insights. In all these cases, verbal insights are matched to the associated video and GPS using G-Code.

^q^We have seen evidence of this around visible crime hotspots, such as a corner store with a group hanging outside.

^r^For a complete overview, see Fontana, A., & Frey, J. (1994). The Art of Science. ‘*The handbook of qualitative research*’ 361–376.
